# Association between the Perioperative Antioxidative Ability of Platelets and Early Post-Transplant Function of Kidney Allografts: A Pilot Study

**DOI:** 10.1371/journal.pone.0029779

**Published:** 2012-01-18

**Authors:** Barbara Dołęgowska, Wojciech Błogowski, Leszek Domański

**Affiliations:** 1 Department of Laboratory Diagnostics and Molecular Medicine, Pomeranian Medical University, Szczecin, Poland; 2 Department of Nephrology, Transplantation and Internal Medicine, Pomeranian Medical University, Szczecin, Poland; University of Colorado, United States of America

## Abstract

**Background:**

Recent studies have demonstrated that the actions of platelets may unfavorably influence post-transplant function of organ allografts. In this study, the association between post-transplant graft function and the perioperative activity of platelet antioxidants was examined among kidney recipients divided into early (EGF), slow (SGF), and delayed graft function (DGF) groups.

**Methodology/Principal Findings:**

Activities of superoxide dismutase, catalase, glutathione transferase (GST), glutathione peroxidase, and glucose-6-phosphate dehydrogenase (G6P) were determined and levels of glutathione, oxidized glutathione, and isoprostane were measured in blood samples collected immediately before and during the first and fifth minutes of renal allograft reperfusion. Our results demonstrated a significant increase in isoprostane levels in all groups. Interestingly, in DGF patients, significantly lower levels of perioperative activity of catalase (p<0.02) and GST (p<0.02) were observed. Moreover, in our study, the activity of platelet antioxidants was associated with intensity of perioperative oxidative stress. For discriminating SGF/DGF from EGF, sensitivity, specificity, and positive and negative predictive values of platelet antioxidants were 81–91%, 50–58%, 32–37%, and 90–90.5%, respectively.

**Conclusions:**

During renal transplantation, significant changes occur in the activity of platelet antioxidants. These changes seem to be associated with post-transplant graft function and can be potentially used to differentiate between EGF and SGF/DGF. To the best of our knowledge, this is the first study to reveal the potential protective role of platelets in the human transplantation setting.

## Introduction

Ischemia-reperfusion injury (I/R) is a very complex phenomenon that may strongly determine both early- and long-term outcomes of transplant recipients. One of the most important components of the I/R process is the intensified oxidative stress that accompanies both phases of I/R. According to recent studies, such temporary systemic oxidative imbalance may trigger a robust inflammatory response within transplanted organ *via* activation of the innate immune system and proinflammatory signaling pathways; it may also lead to cellular destruction due to activation of autophagy or enhancement of chaperone stress [1]–[5].

Platelets are blood elements that are well known as the key components of coagulation processes. However, platelets contribute to additional processes that extend beyond hemostasis and thrombosis. Recent studies revealed that these “cellular fragments” possess sufficient molecular armament that enables them to significantly influence the function of transplanted allografts [6]–[8]. These reports were mainly based on analyses of experimental animal models, in which researchers demonstrated that platelets participate in orchestrating inflammatory responses *via* activation of the CD40/CD154 signaling pathway. Platelets are also able to express several proinflammatory and procoagulation molecules that may finally lead to rejection and destruction of transplanted organs [9], [10]. Several recent studies have highlighted that these observations may also apply to the human solid organ transplantation setting [10]–[12]. However, it is also important to stress that in addition to proinflammatory and procoagulation characteristics, platelets also possess a wide range of protective substances, such as antioxidative enzymes, which could potentially exert a protective influence by limiting the intensity of oxidative stress following I/R injury of various organs [13], [14].

**Figure 1:**
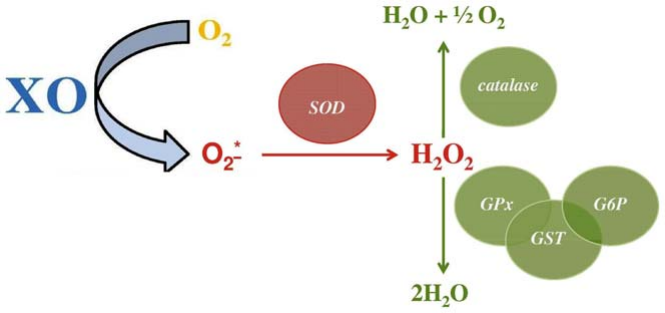
Simplified illustration of enzymatic pathways of superoxide radical dismutation. Superoxide radical, generated by xanthine oxidase activity is neutralized by superoxide dismutase, leading to hydrogen peroxide synthesis. Afterwards, two possible pathways of hydrogen peroxide neutralisation exists – either via catalase activity or via glutathione-related compounds i.e. mainly by glutathione peroxidase that cooperates with glutathione transferase and glucose-6-phosphate dehydrogenase. Glutathione, itself, is the most important “stabiliser” of intracellular oxidative balance. XO – xanthine oxidase, SOD – superoxide dismutase, GPx – glutathione peroxidase, GST – glutathione transferase, G6P – glucose-6-phosphate dehydrogenase, O_2_-^*^ – superoxide radical, H_2_O_2_ – hydrogen peroxide.

In our previous study [15], we demonstrated that during the early phase of kidney allograft reperfusion, higher perioperative activity of prooxidative enzymes, such as xanthine oxidoreductase or oxidase, occurs and that this is strongly associated with development of slow/delayed graft function (SGF/DGF) and poorer 1-year post-transplant allograft function in humans. The activity of these enzymes results in intensified generation of reactive oxygen species, mainly superoxide radical (O_2_-^*^), which may be neutralized by superoxide dismutase (SOD), catalase, or glutathione-related compounds (Figure 1) [13], [14], [16], [17]. However, although platelets possess all necessary enzymes to significantly limit the intensity of oxidative stress during the I/R injury that accompanies renal transplantation, little is known about this function of platelets in the clinical transplantation setting. Therefore, in this study, our purpose was to (i) examine the intensity of oxidative stress and the changes in platelet antioxidants during the first 5 minutes of kidney allograft reperfusion, and (ii) correlate our findings with the clinical outcomes in patients. Our hypothesis was that graft reactivation may be associated with perioperative changes in the activity of platelet antioxidants.

## Materials and Methods

### Ethics statement

This study protocol was approved by the Bioethical Committee of the Pomeranian Medical University in Szczecin, and patients provided informed written consent for participation.

### Patients

This study included 69 consecutive patients, who underwent transplantation in our University center (Department of General and Transplantation Surgery). The patients were divided into 3 groups depending on postoperative graft reactivation: early (immediate), slow, and delayed graft function (EGF, SGF, and DGF, respectively) groups, according to previously described criteria [15], [18], [19]. Patients were assigned to the appropriate study group by nephrologists who had no information regarding the results of molecular parameters. Patients with immediate activation of the graft, defined by serum creatinine levels below 3 mg/dL on the fifth postoperative day, were included in the EGF group. Recipients with graft activation problems were assigned to the SGF (creatinine level higher than 3 mg/dL on the fifth postoperative day, with no requirement of dialysis treatment) or DGF group (need for dialysis in the first week after transplantation). Cross-matching status was determined using a microcytotoxic test (complement-dependent cytotoxicity; CDC). Renal allografts were received from patients who died from craniocerebral trauma (n = 25), subarachnoid hemorrhage (n = 11), intracerebral hemorrhage (n = 19), stroke (n = 5), brain tumor (n = 4), neuroinfection (n = 2), or suicide (n = 3). Grafts were stored cold and perfused with EuroCollins preservation solutions. Allocation of donor kidneys to individual recipients was centrally directed by the Poltransplant system. All patients were undergoing transplantation for the first time and received standard immunosuppressive protocols with triple drug therapy, including cyclosporine A, azathioprine, and steroids. Oral cyclosporine administration was initiated on the day of transplantation at a dose of 5 mg/kg for all patients. On postoperative day 2, patients had received 10 mg·kg^−1^·day^−1^ of cyclosporine. In the case of allograft reactivation problems, this dose was reduced 50%. During follow-up, cyclosporine doses were adjusted to achieve troughs of 200–250 ng/mL for the first 3–6 months, with maintenance levels of 120–180 ng/mL afterwards. The dose of prednisolone was gradually reduced to 5 mg/day over 3–6 months. Acute rejection episodes were defined using Banff criteria and were proven by biopsy. General characteristics of the donors and recipients are summarized in Table 1.

**Table 1:** General characteristics of the donors and recipients (means ± SD).

		EGF	SGF	DGF
***Donors***				
Gender (M-male/F-female)		24 (M-12/F-12)	21 (M-15/F-6)	24 (M-15/F-9)
Age (years)		49±13	50±14*	51±13*
***Recipients***				
Gender (M-men/F-female)		24 (M-10/F-14)	21 (M-17/F-4)**	24 (M-15/F-9)
Age (years)		46±13	48±13	44±11
HD before transplantation (months)		18±12	23±19**	28±16**
Cause of renal insufficiency:				
Glomerulonephritis		13	9	10
ADPKD		3	4	4
arterial hypertension		1	1	2
diabetes		4	4	2
other		3	3	6
Cold ischemia time (hours)		21,85±9,18	20,36±6,48	26,29±10,94#
Time of reperfusion (seconds)		61±8	58±12	61±10
Warm ischemia time (minutes)		4,23±0,55	4,29±0 ,42	4,27±0,63
Surgical revascularization (minutes)		21,35±7,06	23,20±5,64	24,08±5,72
Mismatch (%)	HLA – A	49,80	49,28	50,92
	HLA – B	48,83	47,02	46,33
	HLA – DR	46,12	46,02	46,08
Panel-reactive antibodies (%)		0	0	0
GFR 1 (mL/min)		16±6	13±5	12±4
GFR 5 (mL/min)		39±10	14±5*	14±8*
GFR 10 (mL/min)		51±11	26±13*	18±4**

ADPKD – autosomal dominant polycystic kidney disease EGF – early graft function group HD – hemodialysis.

SGF – slow graft function group DGF – delayed graft function group.

*p<0.05;

**p<0.01 (vs EGF) #p<0.05 (vs SGF).

### Methods

Blood samples were collected perioperatively according to the previously described protocol [20]. Briefly, to determine the dynamics of the platelet antioxidative response, the (0) blood sample was taken from the iliac vein before anastomosing the kidney vessels with the recipient's iliac vessels. Next, once the renal vein of the graft was cannulated, additional blood samples (1) and (5) were taken at the first and fifth minutes of reperfusion. Reperfusion of the transplanted kidney was monitored using the ThermaCAM SC500 (AGEMA, Infrared System AB, Danderyd, Sweden) thermovision camera, which detects infrared radiation and records digital images showing the surface temperature distribution of tested objects. The process of total reperfusion was completed when the scans from the thermovision camera demonstrated that the entire organ was filled with the recipient's blood.

### Assays of platelet antioxidants

For quantitative assessment of changes in platelet antioxidative response, 5 mL of blood was collected and mixed with 109 mM trisodium citrate (9∶1; v/v). Blood was centrifuged (10 min; 20°C; 106× *g*), and platelet-rich plasma (PRP) was obtained. PRP was then transferred to a new tube and re-centrifuged (10 min; 20°C; 3824× *g*). Platelet-poor plasma (PPP) was transferred to a new tube and stored at −80°C until assessment was performed. Platelet sediments were washed twice in Tyrode buffer (pH 7.4) and then suspended in it (1 mL). Afterwards, platelets were frozen and stored according to previously published recommendations [21], [22].

Platelet lysates were obtained by adding Triton X-100 (0.1%) to defrosted platelet suspensions. Platelet levels of SOD, catalase, glutathione peroxidase (GPx), glutathione transferase (GST), glucose-6-phosphate dehydrogenase (G6P), and reduced and oxidized glutathione (GSH and GSSG, respectively) were measured using spectrophotometry according to previously described methods [14], [15], [23].

### Assays of systemic oxidative stress intensity

Plasma isoprostane (8-iPF_2α_-III) levels were determined by the ELISA method using the BIOXYTECH® 8-Isoprostane Assay Kit reagent set (OxisResearch®, USA) according to the manufacturer's recommendations and previously described protocols [24], [25].

### Clinical parameters of allograft function

To determine allograft function, creatinine levels were measured during perioperative periods (i.e., on the first, fifth, and tenth post-transplant day) and during follow-up visits during the first year after transplantation.

### Clinical predictive value of examined parameters

Application of cut-off limits enabled classification of patients into 4 categories: true positive, true negative, false positive, and false negative. Sensitivity, specificity, and positive and negative predictive values were calculated.

### Statistical analysis

To determine the distribution of variables, Shapiro–Wilk's test was used. For comparison of the mean parameter values between the examined groups, Student's t-test was used (for normally distributed variables). Abnormally distributed variables were log transformed. If normal distribution was achieved, variables were also compared using Student's t-test. However, if this transformation did not change the distribution, the Mann-Whitney test was performed. Differences between concentrations of analyzed parameters in consecutive minutes of reperfusion were assessed by Friedmann's ANOVA test. To evaluate the effect(s) of continuous variables on perioperative intensity of oxidative stress, multivariate regression analyses were performed with a stepwise selection method. Variables excluded from the initial model were re-entered individually to exclude residual confounding. During development of multivariate regression models, the number of inserted independent variables did not exceed 10% of the total number of analyzed patients. Constructed models were verified using the Akaike information criterion (AIC), and wrongly constructed matrices resulted in rejection of the model. Receiver operating characteristics (ROC) curves were constructed for all analyzed parameters as diagnostics for graft activation problems (i.e., SGF and DGF), and the area under each ROC curve (AUC) was calculated. Statistical analysis was performed using SPSS statistical analysis software. Statistical significance was defined when p values were less than 0.05.

## Results

**Figure 2:**
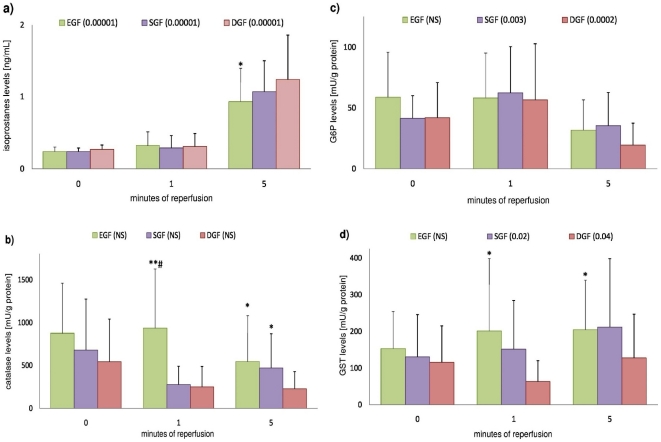
Mean isoprostanes, catalase, G6P and GST values stated in consecutive minutes of graft reperfusion, and statistical comparison of these means between examined groups. (A) Isoprostanes, (B) Catalase, (C) G6P and (D) GST. EGF – early graft function, SGF – slow graft function, DGF – delayed graft function group, GST – glutathione transferase, G6P – glucose-6-phosphate dehydrogenase. P values in parentheses represent result of Friedmann's ANOVA test for appropriate group. *p<0.05; **p<0.01 – level of significance for differences between means (vs DGF). #p<0.05 – level of significance for differences between means (vs SGF).

The mean values of examined parameters and comparisons between the analyzed groups are presented in Figure 2 and Table S1.

### Intensity of systemic perioperative oxidative stress

Statistical analysis revealed no significant differences between examined groups in terms of intensity of oxidative stress immediately before allograft reperfusion. However, during the reperfusion period, significant increases in isoprostane levels were observed in all analyzed groups. At the fifth minute of reperfusion, isoprostane concentrations were significantly higher in DGF patients than in EGF patients (Figure 2). No statistically significant differences were observed between EGF and SGF patients.

### Analysis of the antioxidative armament of platelets

Statistical analysis of the mean activity/concentrations of platelet antioxidants measured immediately before allograft reperfusion revealed no significant differences between the examined groups. In our study, we could not demonstrate statistically significant changes among reperfusion times or differences among analyzed groups in terms of SOD levels (Table S1).

Relatively analogical tendencies were observed in terms of changes in levels/activity of selected platelet glutathione-related antioxidants. Namely, GSH, GPx, and GSSG activity/levels were comparable between the examined groups throughout the analyzed perioperative period and did not significantly change within reperfusion time, with the exception of GSSG levels that significantly decreased during the analyzed reperfusion period in all groups (Table S1).

Moreover, although stable action of G6P was observed throughout the analyzed reperfusion period in EGF patients, in patients with allograft activation problems, the mean activity of this antioxidant significantly decreased during the reperfusion time (p = 0.003 and p = 0.0002 for SGF and DGF respectively). Our analysis also demonstrated that although perioperative GST activity significantly increased in SGF and DGF patients, GST activity was significantly lower in DGF individuals than in EGF individuals (p<0.02).

Finally, although no statistically significant changes in catalase activity were observed during the reperfusion time, mean activity of this antioxidant was constantly significantly lower in DGF patients (and also partially in SGF patients) than in EGF patients (Figure 2).

### Perioperative activity/levels of platelet antioxidants and intensity of systemic oxidative stress

To verify whether an association exists between the perioperative activity/levels of the platelet antioxidative system and intensity of systemic oxidative stress, we performed a pilot multivariate analysis with isoprostane levels as dependent variables (representative models presented in Table 2). These results demonstrated several strong negative associations between activity/levels of platelet antioxidative compounds and perioperative isoprostane concentrations. To our surprise, in multivariate regression analysis, various clinical parameters, such as donors' or recipients' age, (terminal) creatinine level, duration of cold and warm ischemia time, or hemodialysis treatment before transplantation were not “directly” associated with perioperative oxidative stress intensity in any group; however, some of these parameters along with platelet antioxidants influenced “general” post-transplant allograft function (Table 2).

**Table 2:** Pilot results of representative multivariate linear regression models.

AnalyzedGroup	Dependentvariable	Independentvariable	β	P of the variable	R^2^	P of the model
***All groups***	Allograft reactivation*	catalase (1)	−0.37	0.04	0.59	0.004
		CIT	0.40	0.02		
		catalase (5)	−0.41	0.03		
		HD-pre-Tx	0.15	0.04		
***EGF group***	iP(0)	GPx(0)	−0.93	0.02	0.87	0.02
	iP(1)	GST(0)	−0.68	0.008	0.87	0.005
		catalase(1)	−0.66	0.007		
		GPx(1)	−0.44	0.04		
	iP(5)	G6P(1)	−0.75	0.01	0.56	0.01
***SGF group***	iP(0)	GPx(0)	−0.77	0.02	0.60	0.02
	iP(5)	GSSG(5)	0.83	0.002	0.59	0.005
		catalase(1)	−0.61	0.01		
***DGF group***	iP(1)	G6P(0)	−0.53	0.03	0.23	0.03
	iP(5)	SOD(1)	−0.97	0.00001	0.84	0.00002
		GSH(5)	−0.99	0.00004		
		GST(0)	−0.65	0.004		

(0), (1), (5) – consecutive analyzed minutes of reperfusion.

G6P – glucose 6-phosphate dehydrogenase GPx – glutathione peroxidase.

GST – glutathione transferase SOD – superoxide dismutase.

GSH – reduced glutathione GSSG – oxidized glutathione.

CIT – cold ischemia time iP – isoprostanes.

HD-pre-Tx – duration of hemodialysis treatment prior to transplantation.

β – standardized coefficient in the regression equation p – level of significance.

*EGF, SGF and DGF were assigned 0, 1 and 2 value (respectively); variables excluded from the model: GST(5) and GPx(5) – in both cases P>0.05.

### Clinical value of platelet anti-oxidants to discriminate SGF/DGF from EGF

In order to establish whether perioperative changes in the activity/levels of platelet antioxidants may serve as novel markers/predictors of post-transplant graft function, we constructed ROC curves and calculated approximate AUC values. Among all clinical and newly examined parameters, only those with the highest AUC and 95% CI lower bound value are precisely described (Table 3). For each parameter, the appropriate “suggested” cut-off value was applied; this offered the highest clinical sensitivity and specificity.

**Table 3:** Diagnostic value of examined parameters to discriminate delayed and slow allograft function from immediate allograft function.

Parameter	catalase (1)	catalase (5)	G6P (0)
Area under curve and p value	0.83; P<0.001	0.72; p<0.03	0.72; P<0.03
Suggested cut-off value [mU/mL]	≤257,72	≤336,10	≤48,27
Sensitivity [%]	91	81,8	81,8
Specificity [%]	55,3	50,0	57,9
Positive predictive value [%]	37	32,1	36
Negative predictive value [%]	95,5	90,5	91,7

G6P – glucose-6-phosphate dehydrogenase.

(0), (1), (5) – consecutive analyzed minutes of reperfusion.

p – level of significance.

## Discussion

In various studies, it has been reported that intensive perioperative oxidative stress occurring during kidney transplantation may contribute to development of post-transplant complications and that this stress may be limited by antioxidant supplementation during renal I/R injury [26]–[30]. Recent preliminary reports suggest that platelets exert profound influences on the function of transplanted cells/organs and may independently contribute to allograft rejection [8]–[10]. Nevertheless, only a few studies have addressed the biochemical influence of platelets on kidney allograft function in the human transplantation setting. Therefore, in this study, we decided to verify the effects of perioperative antioxidative responses of platelets on post-transplant renal allograft function in humans.

In our study, we observed several significant differences in platelet antioxidative activity, which varied according to the patient's post-transplant outcome. Namely, we noticed that both platelet pathways of hydrogen peroxide neutralization (*via* catalase or glutathione-related compounds) seem to be less efficient in patients with post-transplant graft reactivation problems (SGF/DGF) than in EGF patients. Lower activity of catalase and GST was observed in the SGF/DGF groups; these groups also showed significant lowering of G6P action during the reperfusion period. Several recent papers have demonstrated that excessive free radical generation promotes vasoconstriction and plays a key role in the development of renal injury that may be prevented by over-expression of catalase, which modulates the intrarenal renin-angiotensin system [31], [32]. Our study indirectly supports these observations, as catalase activity was strongly associated with post-transplant kidney allograft function and perioperative activity of catalase was significantly lower in patients having problems with postoperative allograft reactivation.

Platelet antioxidative enzymes were negatively associated with perioperative systemic isoprostane levels; concentrations of these enzymes significantly increased during the reperfusion time in all groups and were higher in DGF individuals in the fifth minute of reperfusion. Thus, our experiments highlight that during renal transplantation, intensified oxidative stress always occurs; however, this stress is most evidently pronounced in DGF individuals. Moreover, we hypothesize that during the early phase of kidney allograft reperfusion, platelet antioxidants probably exert a protective effect by modulating the perioperative intensity of oxidative stress that accompanies the reperfusion period following renal transplantation. This potential protection offered by platelets seems to be less expressed in SGF/DGF patients and probably clinically translates to poorer post-transplant allograft function. However, the results of our study are based on a relatively small number of patients and may be underpowered. In order to fully verify the proposed hypothesis and derive definite conclusions, further studies are required. These studies could benefit from our analysis, which offers valuable information on the appropriate sample size necessary for reliable verification of the observed phenomena. In addition, other plausible clinical factors that could be responsible for observed tendencies must also be taken into account, including organ quality, which may increase the risk of poorer post-transplant allograft function.

The results of our study support previous observations that clinical parameters such as duration of cold ischemia time (CIT) and/or pre-transplant hemodialysis treatment may unfavorably influence early post-transplant allograft function [33]; however, we also demonstrated that this effect does not seem to be “directly” associated with the modulation of perioperative intensity of oxidative stress (as no statistical interactions were observed between these clinical parameters and isoprostane levels). We speculate that this tendency is observed because prolonged duration of ischemic time stimulates upregulation of various pro- and antioxidative enzymes, rather than generating free radicals. In our recent paper, we have demonstrated that during kidney I/R injury, upregulation of xanthine oxidoreductase (XOR) activity occurs and is most pronounced in patients with the longest CIT values [16]. Nevertheless, although we have established a direct association between XOR activity and intensity of isoprostane levels *(unpublished observation)*, in the present study, we did not observe a correlation between CIT and isoprostane concentrations. Therefore, our results suggest that while the influence of CIT on modulation of postoperative allograft function is inherent, its effect on perioperative changes in oxidative stress intensity seems to be indirect and probably modulated by the recipient's antioxidative potential.

Finally, the clinical value of platelet antioxidant activity/levels as diagnostic markers for SGF/DGF prediction must be emphasized. In our study, we have demonstrated that several platelet antioxidants may help differentiate between EGF and SGF/DGF. Among these parameters, catalase and G6P activity seems to be of the greatest diagnostic value for SGF/DGF prediction. Thus far, several authors have discovered clinically suitable early post-transplant markers for SGF/DGF prediction [34], [35]; however, our team concentrates on finding pre- and/or perioperative SGF/DGF predictors [16], [19], [20], [36]. In the current study, we described novel pre- and perioperative markers (activity of platelet G6P and catalase, respectively) that have a sensitivity of up to 80–90% and therefore seem to be promising tools for prediction of post-transplant outcome in the future. Unfortunately, at this stage of our research, these markers do not seem suitable for independent decision making because of their low specificity. Moreover, their cut-off and predictive values must be verified in further cohort studies.

Nevertheless, our study demonstrated that the early phase of kidney allograft reperfusion is associated with intensified oxidative stress and is accompanied by significant changes in the activity/levels of platelet antioxidants, whose actions may contribute to limitation of the perioperative intensity of oxidative stress. Our study also highlighted the lack of “full” activity of platelet antioxidative armament as a potential biochemical explanation for problems associated with post-transplant graft activation. Finally, we also demonstrated that the perioperative activity of selected platelet antioxidants may help differentiate between EGF and SGF/DGF.

## Supporting Information

Table S1 **Examined parameters' values measured in consecutive minutes of graft reperfusion, and the statistical analysis of these means between the examined groups (means ± SD).**
(DOC)
