# Supplementary material for: Association between the Perioperative Antioxidative Ability of Platelets and Early Post-Transplant Function of Kidney Allografts: A Pilot Study
Source: PLoS One. 2012 Jan 18;7(1):e29779. doi: 10.1371/journal.pone.0029779 (PMC3261166; doi:10.1371/journal.pone.0029779)
Supplement: Table S1 — Examined parameters' values measured in consecutive minutes of graft reperfusion, and the statistical analysis of these means between the examined groups (means ± SD). (DOC) [file pone.0029779.s001.doc]

**Table S1. Examined parameters` values measured in consecutive minutes of graft reperfusion, and the statistical analysis of these means between the examined groups (means ± SD).**

**Parameter/minutes 0 1 5 Friedmann`s ANOVA test (p)**

**SOD** [mU/g protein]

*EGF* 2851,32 ± 1682,12 4005,32 ± 2461,58 3412,46 ± 2270,14 0.31

*SGF* 2406,24 ± 1482,56 3716,51 ± 2807,86 2811,50 ± 2013,84 0.27

*DGF* 2284,24 ± 2255,68 3062,91 ± 2379,85 2412,96 ± 2159,83 0.09

**GSH** [µmol/g protein]

*EGF* 42,35 ± 21,10 41,17 ± 24,10 52,02 ± 18,18 0.09

*SGF* 30,93 ± 15,30 35,32 ± 21,24 35,83 ± 22,66 0.94

*DGF* 34,23 ± 23,10 41,63 ± 36,17 46,22 ± 40,57 0.08

**GSSG** [mU/g protein]

*EGF* 1354,16 ± 676,48 759,05 ± 744,42 299,55 ± 136,37 0.003

*SGF* 884,37 ± 589,29 466,69 ± 275,90 196,77 ± 176,49 0.00001

*DGF* 1160,56 ± 907,39 564,03 ± 473,18 327,93 ± 311,95 0.0001

**GPx** [mU/g protein]

*EGF* 143,68 ± 110,53 159,46 ± 117,16 199,64 ± 152,58 0.18

*SGF* 156,64 ± 126,92 109,85 ± 90,09 205,54 ± 170,96 0.08

*DGF* 91,00 ± 88,84 106,79 ± 98,75 145,07 ± 131,07 0.21

EGF – early graft function group SGF – slow graft function group DGF – delayed graft function group

SOD – superoxide dismutase GSH – reduced glutathione GSSG – oxidized glutathione

GPx – glutathione peroxidase p – level of significance

no statistically significant differences between analyzed groups were stated in comparison of mean values of platelets anti-oxidants` activity
